# Editorial: Calcium pyrophosphate deposition disease

**DOI:** 10.3389/fmed.2025.1557035

**Published:** 2025-01-30

**Authors:** Georgios Filippou, Nicola Dalbeth, Sara K. Tedeschi

**Affiliations:** ^1^Department of Rheumatology, IRCCS Galeazzi – Sant'Ambrogio Hospital, Milan, Italy; ^2^Department of Biomedical and Clinical Sciences, University of Milan, Milan, Italy; ^3^Department of Medicine, University of Auckland, Auckland, New Zealand; ^4^Division of Rheumatology, Inflammation and Immunity, Brigham and Women's Hospital, Boston, MA, United States

**Keywords:** chondrocalcinosis, CPPD, CPP crystal deposition disease, crystal arthropathies, calcium pyrophosphate, calcium pyrophosphate arthropathy

Calcium Pyrophosphate Deposition (CPPD) appears to be a common condition among the elderly, though the exact prevalence and incidence are not known (1). CPPD is an umbrella term that encompasses all instances of calcium pyrophosphate (CPP) crystal deposition in joints and periarticular tissues. The condition can be asymptomatic and discovered incidentally, or can lead to various forms of arthritis, ranging from acute flares generally occurring in a large joint to a chronic polyarticular arthritis that resembles rheumatoid arthritis (2).

The journey from the discovery of this condition to the current state of knowledge has been neither straightforward nor consistent, marked by prolonged periods of limited research activity. As Pineda et al. observe in their historical overview, over a century elapsed from the first observation of chondrocalcinosis to McCarty's identification of calcium pyrophosphate crystals in 1960. However, the past decade has witnessed a resurgence of interest in CPPD, as evidenced by the exponential increase in publications in this field.

One of the main drivers of this renewed interest in CPPD has undoubtedly been imaging. Modern imaging such as ultrasound has complemented traditional approaches such as conventional radiology, and collaborative research efforts have refined the roles of these methods (3, 4). This progress has paved the way for innovative imaging approaches. Ulas et al. applied the four dimensional computed tomography to examine the wrist of patients with CPPD, an area that is very frequently involved by the disease and is difficult to examine with standard two dimensional imaging due to its intricate anatomy and biomechanics. The results provide interesting insights and set the stage for future studies. Similarly, the application of artificial intelligence in CPPD diagnosis holds promise. Hügle et al. tested a deep learning model for identification of CPPD in hand/wrist radiographs yielding compelling results. Widespread adoption of such models could enhance diagnostic accuracy and minimize misdiagnoses, while also increasing physician awareness of the high prevalence of CPPD.

Despite the strides in CPPD diagnosis, effective treatment remains elusive. No drugs are specifically approved for CPPD arthritis, and no substances have demonstrated the ability to reduce CPP crystal burden. Voulgari et al. provided an extensive and up-to-date review of available treatments, emphasizing the pressing need for new clinical trials and targeted therapies. Porta et al. explored the efficacy of a high molecular weight hyaluronic acid combined with a collagen tripeptide in patients with osteoarthritis (OA) and CPPD. This phenotype poses unique challenges due to the accelerated and severe joint damage caused by amplified inflammation from CPP crystals. The compound showed promise in alleviating pain in knee OA and CPPD patients.

Finally, a significant barrier to advancing CPPD research lies in the lack of *in vivo* models to investigate the condition's pathogenesis. As Luisetto and Scanu highlight, attempts to develop genetically modified mouse models to replicate calcification processes have thus far been unsuccessful. Current basic research relies on crystal-induced inflammation models, which primarily explore inflammatory pathways but fail to elucidate CPPD pathophysiology.

In conclusion, CPPD is a complex condition that has recently rekindled the interest of researchers. This has led to some significant advances in the diagnosis and definition of the disease as demonstrated by the development of imaging recommendations (4) and the CPPD disease classification criteria (3) but a much work still remains, as treatment options are limited and basic research is hindered by the lack of animal models. The CPPD timeline outlined by Pineda et al. stretches into an uncertain future, with much work to be done to fully understand and effectively treat this complex condition.
